# Beliefs about schizophrenia’s causes among family members

**DOI:** 10.1192/j.eurpsy.2021.1381

**Published:** 2021-08-13

**Authors:** I. Lajmi, S. Omri, N. Smaoui, R. Feki, J. Ben Thabet, M. Maalej Bouali, L. Zouari, N. Charfi, M. Maalej

**Affiliations:** Psychiatry C Department, Hedi chaker University hospital, sfax, Tunisia

**Keywords:** schizophrenia’s causes, family members

## Abstract

**Introduction:**

Investigating family members’ causal beliefs regarding schizophrenia is an important step in the management of the illness; it may affect adherence to treatment of patients with schizophrenia.

**Objectives:**

To investigate the beliefs about the causes of schizophrenia among families of patients with schizophrenia.

**Methods:**

We led a cross sectional descriptive study involving 32family members of patients suffering from schizophrenia who were followed in the psychiatry department of Hedi Chaker University Hospital in Sfax (Tunisia), between May to September 2019. Data was collected from the medical records and the questionnaire designed to the study.

**Results:**

The mean age of the relatives was 60.8 years, with a sex-ratio of 1.6. The relatives included 20 fathers (62.5%), six wives (18.8%), three mothers (9.4%), and three sisters (9.4%). Among them, 37.5 % were illiterate; 37.5% lived in rural area. Fourteen participants (43.8%) had cited at least two possible causes of schizophrenia. The most frequently cited causes were emotional shock (81%) and god’s will or fate (72%) followed by witchcraft (37.7%). The belief of God’s will and fate was associated with poor compliance to treatment (p=0.06). The belief of supernatural cause was correlated to rural origin (p=0.000) and advanced age of participants (p=0.000).Significant correlation was also found between family history of mental disorders and beliefs on hereditary causes (p=0.000).

**Conclusions:**

These results suggest that family members of patients suffering from schizophrenia need to be better informed about its main causes to improve both compliance to treatment and social integration of this patients.

